# Research productivity on spontaneous intracranial hypotension: A bibliometric analysis

**DOI:** 10.1016/j.bas.2024.103324

**Published:** 2024-08-30

**Authors:** Christopher Marvin Jesse, Nicolas W. Graf, Levin Häni, Johannes Goldberg, Tomas Dobrocky, Eike I. Piechowiak, Andreas Raabe, Ralph T. Schär

**Affiliations:** a Department of Neurosurgery, Inselspital, Bern University Hospital, and University of Bern, Bern, Switzerland; b Institute of Diagnostic and Interventional Neuroradiology, Inselspital, Bern University Hospital, and University of Bern, Bern, Switzerland

**Keywords:** Spontaneous intracranial hypotension, SIH, Spontaneous cerebrospinal fluid leak, Cerebrospinal fluid hypovolemia, Publication research productivity

## Abstract

**Introduction:**

Spontaneous intracranial hypotension (SIH) is an important cause of devastating headaches and caused by CSF-leaks in the spine.

**Research question:**

The aim of this analysis was to gain an overview of the progress of research on SIH over time. The global publication landscape relating to SIH was analyzed and comparisons between regions were made.

**Material and methods:**

A bibliometric analysis was performed by searching for research articles on SIH in PubMed published between 1983 and 2022. Countries responsible for the publications were ranked by the sum of citations. An average annual growth rate was calculated and the density of SIH publications per 100 000 physicians was determined.

**Results:**

We identified 974 articles. In 1983 only one SIH patient was reported; in 2021 the number of patients had increased to 4230. The average annual growth rate of SIH publications during this period was 12.7%. The most common publication type were case reports (n = 570). The most common medical specialty of the first author was neurology (n = 251) followed by neurosurgery (n = 250) and radiology (n = 191). Although most publications originated from the United States of America (USA), South Korea had the highest density of SIH investigators (37.86 publications per 100 000 medical doctors). The most cited paper (296 citations) was published in 2006 in *JAMA* (USA).

**Discussion and conclusion:**

Research on SIH has increased exponentially over the past four decades. The international community of SIH researchers is growing, and with it the opportunities for global networks involved in research, treatment, and patient education.

## Highlights

• Research on SIH has increased significantly (average annual growth rate: 12.7%).
• The most common type of publication on SIH is case reports (58.5%).
• United States produced the highest number of publications.
• Most frequent specialties of first author were neurology, neurosurgery and radiology.
• The most cited paper on SIH was published in 2006 in JAMA, receiving 296 citations.

## 1 Introduction

Spontaneous intracranial hypotension (SIH) is primarily caused by cerebrospinal fluid (CSF) leaks in the cervicothoracic spine. It has an estimated incidence of 4–5 cases per 100 000 population per year (Schievink, 2006; Schievink et al., 2007, 2022). SIH has a notable negative impact on the social and working life of affected patients as well as on health-related quality of life (Jesse et al., 2022). Although orthostatic headaches are the hallmark symptom of SIH, the condition gives rise to a range of clinical manifestations, frequently escaping accurate diagnosis (D'Antona et al., 2021; Schievink, 2000; Hoffmann and Goadsby, 2013; Jesse et al., 2024).

The first description of SIH dates back to 1948 (DISCUSSION ON INTRACRANIAL HYPOTENSION [Abstract], 1948). Although the disease may still be underdiagnosed, awareness about SIH in the medical community has increased. This has resulted in a surge in diagnosed cases and published literature about the condition. Some authors suggest that this is primarily attributable to advancements in medical imaging techniques. (Schievink, 2006)^,^ (Fichtner et al., 2016) A quick search for the term SIH in the online meta database *PubMed* (National Library of Medicine, Bethesda, MD, USA) yielded 1154 results. Diagnostic imaging guidelines on the disease have recently been proposed (Cheema et al., 2023; Dobrocky et al., 2019; Pinto et al., 2023; Relationship of Bern Score; Brinjikji et al., 2021). The number of publications globally is increasing each year (Johnson et al.). Despite this, and the fact that extensive research on SIH is being carried out, no comprehensive bibliometric analysis of SIH articles has been conducted to date. Objective quantification of authors and publication origins is essential for advancing coordinated research efforts and improving guidelines for diagnosis and treatment. Given that there are articles on SIH dating back more than 70 years, (DISCUSSION ON INTRACRANIAL HYPOTENSION [Abstract], 1948; Raskin, 1990) the analysis and quantification of existing research is long overdue.

The aim of this article is thus to present a detailed synopsis of the current state of research articles published on this topic. With our bibliometric analysis on SIH we intend to simplify the comparison and comprehension of research on SIH from different countries. We want to stimulate the dialogue in the scientific community and facilitate efforts towards unification of guidelines.

## 2 Material and Methods

The following section headings are used in accordance with the latest PRISMA guidelines for systematic reviews (Page et al., 2021).

### 2.1 Study design

We conducted a bibliometric analysis on the changing trends of publications on SIH. The requirement for ethical approval was waived due to the study design.

### 2.2 Eligibility criteria and selection process

The inclusion and exclusion criteria were applied by author NG: Only articles available online on PubMed were included. For better comparability, and to rule out duplicates in different languages, only articles written in English were included. The following were excluded: i) articles without an abstract or access to the text, ii) article types “comment” and “erratum”, and iii) articles for which access to credentials for the authors (e.g., the institutions to which they were affiliated) was not possible through the PubMed “affiliations” section or manually through full text access. Articles that were not available online on PubMed were beyond the scope of this study and were therefore excluded.

### 2.3 Information sources

The National Library of Medicine (NLM)'s PubMed served as the data source. We performed our search on April 1, 2022; therefore, the cutoff-date for the study was March 31, 2022.

### 2.4 Search strategy

We applied the following search terms: “spontaneous intracranial hypotension” or “spontaneous cerebrospinal fluid leak” or “cerebrospinal fluid hypovolemia” or “cerebrospinal fluid hypovolemia syndrome” or “hypoliquorrhea” or “spontaneous spinal cerebrospinal fluid leak”.

We decided to multiply the results for the count of publications in 2022 by four to make that year's results comparable with the previous years.

### 2.5 Data collection process

Each article was manually screened on PubMed by NG, and the following information was extracted: Article title, date of publication, names of first author (FA), country of affiliation of FA, medical specialty of FA, institutional affiliation of FA, journal, number of citations in PubMed, publication type and the number of patients studies. Data regarding last authors (LA) is available upon request and can be found in Suppl. Tables 4 and 5 Data were entered in Microsoft Excel version 16.73, 2023 (Microsoft Corporation, Redmond, WA, USA). The number of citations was obtained using the information provided by PubMed in the section “cited by".

Additional data were downloaded externally to sort the articles further: The Journal Impact Factors were retrieved on 2 August 2022 via the Journal Citation Reports of Clarivate (Clarivate Analytics, Philadelphia, PA, USA) (Clarivate, 2022). The density of physicians per 10 000 population was derived from the World Health Organization (WHO, Geneva, Switzerland) on April 16th, 2023 (World Health Organization (WHO), 2023). The WHO only provides records of the density of physicians for each country. Therefore, the absolute number of physicians in each country was calculated by dividing the population of each country in 2021 by the number of physicians per 10 000 population reported by the WHO. The required population data were retrieved from Our World in Data, (Global Change Data Lab, England and Wales) on April 19th, 2023 (Roser et al., 2021).

### 2.6 Ranking of results

Authors, countries, and medical institutions were ranked according to the sum of their citation counts and occurrences on PubMed in our search. Unless otherwise stated, all our rankings apply to FAs and/or LAs. The density of publications per 100 000 physicians was calculated as follows: The count of FAs publishing articles on SIH from each country was divided by the overall population of physicians in that country. This ratio was then multiplied by 100 000 and sorted from the largest to smallest number. Data are shown as descriptive data. No additional statistical analyses were needed.

### 2.7 Calculation of growth rate

To calculate the average publication growth rate, we used the formula for compound annual growth rates (CAGR).

$$CAGR\;\left(t_{0},t_{n}\right)={\left(\frac{V\left(t_{n}\right)}{V\left(t_{0}\right)}\right)}^{\frac{1}{t_{n}-t_{0}}}-1$$

Where $V\left(t_{n}\right)$ represents the sum of publications in 2021, $V\left(t_{0}\right)$ the number of publications in 1983, and $t_{n\;}-t_{0\;}$ the number of years.

## 3 Results

Our literature search resulted in a total of 1154 articles. After application of the above-mentioned exclusion criteria, 974 articles remained.

### 3.1 Analysis per year

**Fig. 1:**
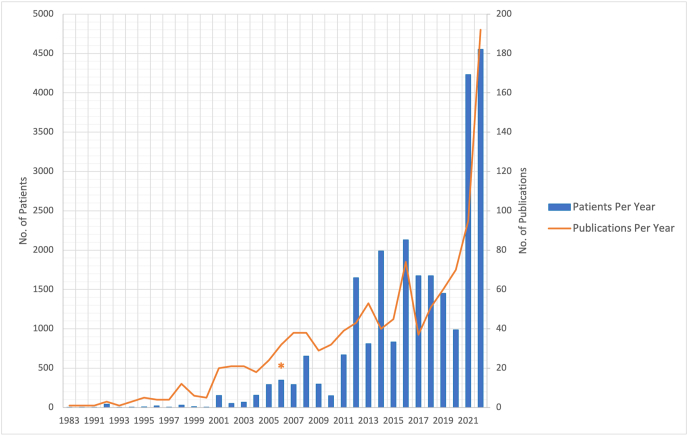
Annual overview of patients included in the SIH research analyzed and count of articles published on SIH. Numbers for 2022 are estimates, based on values for the first quarter of 2022 multiplied by four. Doublings are possible. The asterisk indicates the year in which the most cited SIH paper was published.

From the data collected, a clear uptrend was evident. The first year for which a publication could be found online was 1983 and only one article was published that year. Almost forty years later this number had increased to 95 (2021). This was also the most productive year since the first publication. The uptrend is likely to continue although it is worth noting that there were less productive years too (see Fig. 1). The average yearly growth rate between 1983 and 2021 was 12.7%.

Along with the surge in publications the number of reported patients also increased. In 1983, the year of the first publication on PubMed, only one patient was studied; twenty years later that number had increased to 69. In 2013 there were 812 patients and in 2020 the number of patients had increased to 4230 (Fig. 1).

### 3.2 Ranking by publication type

Of the 974 publications, most (n = 570; 58.5%) were case reports (CR). Two hundred and six different journals published CR. As seen in Table 1, thirty-nine is the highest number of CR published in the same journal. Original articles (OA) accounted for 24.9% of all articles.

**Table 1:** Distribution of most popular publication type (case report) amongst journals.

PUBLICATION TYPE	COUNT OF PUBLICATION TYPE	PERCENTAGE
CR	570	58.5
OA	243	24.9
RV	112	11.5
Letter	23	2.4
Comparative Study	9	0.9
Editorial	8	0.8
Meta-Analysis	2	0.2
Book	1	0.1
Conference Paper	1	0.1
Practical Note	1	0.1
Other	1	0.1
Technical Video	1	0.1
Journal Club	1	0.1
Opinion Statement	1	0.1
Grand Total	974	100.0

CR = Case Report; OA = Original article; RV = Review.

Ninety-four different journals published OA. Most of them (n = 28, 11.5%) were published in the *American Journal of Neuroradiology* (American Society of Neuroradiology, Oak Brook, IL, USA) and 14 (5.8%) each in *Neurology* (Wolters Kluwer on behalf of the American Academy of Neurology, Minneapolis, MN, USA), the *Journal of Neurosurgery* (American Association of Neurological Surgeons, Rolling Meadows, IL, USA) and *Cephalalgia: An International Journal of Headache* (Sage Publishing, Thousand Oaks, CA, USA).

Reviews were published in 62 different journals. The highest number of reviews published in the same journal was eight *Current Pain and Headache Reports* (Springer Science + Business Media, Berlin/Heidelberg, Germany). The first review on SIH was published in 1995. Since then, there has been an uptrend in the publication of reviews with the sum rising to nine in the first quarter of 2022.

### 3.3 Ranking by specialty

**Fig. 2:**
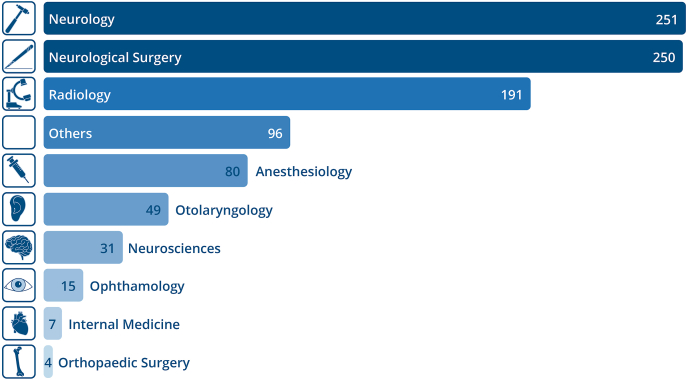
The most common medical specialties of first authors publishing articles on SIH between 1983 and the first quarter of 2022. Radiology and neuroradiology were merged due to unclear differentiation.

Most of the papers’ first authors (FAs) affiliation was listed as a department or institute specializing in neurology (25.8%) or neurological surgery (25.7%). Radiology ranked third (19.6%). Overall, the FAs of 9.9% of publications had a specialty that was not one of the most common ones, e.g., obstetrics and gynecology or pediatrics, or no clear allocation was possible. These cases were labeled “others” (Fig. 2).

### 3.4 Analysis per country

**Fig. 3:**
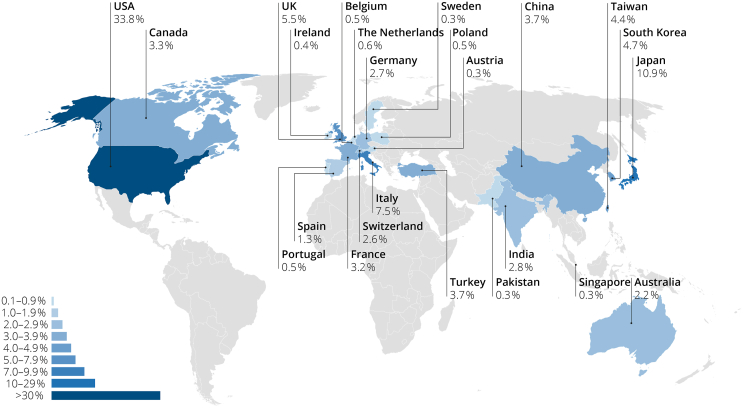
Global distribution of published articles from first authors on SIH between 1983 and 2022.

The countries of origin of the published articles were diverse with FAs’ affiliations in more than 50 countries. The most represented are shown in Fig. 3.

**Fig. 4:**
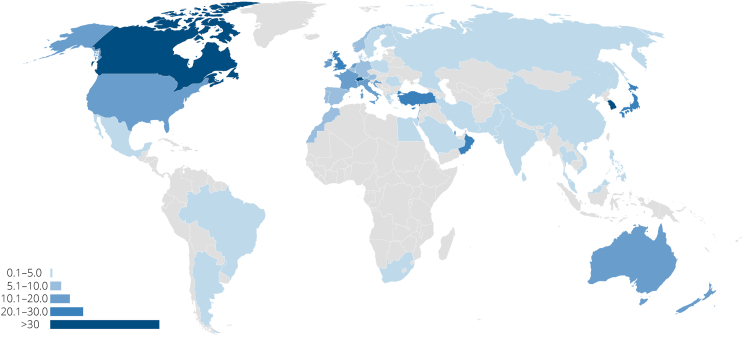
Publications on SIH per 100 000 physicians. Data is based on first author-count and correction with the number of physicians per 10 000 population provided by the World Health Organization and the population count of each country provided by Our World in Data.

Physicians in the USA contributed 33.8% of the total articles. The USA was responsible for 25% of all publications in 2001 and had increased its share to 43% in 2021. As shown in Fig. 3 and Suppl. Table 1, the highest number of publications originated from the USA, followed by Japan and Italy. Applying the correction for absolute number of physicians changed the distribution of publications (see Suppl. Table 2). After correction, South Korea had the highest density of published articles on SIH per physician, followed by Canada and Switzerland (Fig. 4).

An overview of the most common specializations in different countries can also be found in Suppl. Table 3.

#### 3.4.1 North America and the United States

The USA and Canada accounted for 37.1% of all publications: 329 of the 974 articles originated from the USA (see Suppl. Table 1). The most frequently listed specialty regarding author affiliation in the US was neurosurgery which accounted for 33.4% of all FAs from the US publishing on SIH. Seventy-one of the FAs were based in California, eight in New York, and six in Minnesota. Eighty-one radiologists, mostly based in North Carolina (n = 24), 14 in Minnesota, and ten in California, participated in SIH research. Fifty-three neurologists did so, too. Fourteen of were based in Minnesota, five in Texas and four in Arizona.

The medical centers with which the highest numbers of FAs were affiliated – both within the USA and globally – were Cedars-Sinai Medical Center in Los Angeles, CA, the Duke University Medical Center in Durham, NC, and the Mayo Clinic in Rochester, MN.

#### 3.4.2 Asia

Twenty-five percent of the FA reported affiliations in Asian countries. Of the articles from Asia, Japan published the most. South Korea, Taiwan Republic of China (ROC), the People's Republic of China, and India followed. After rank correction, South Korea had the highest density of articles published on SIH per investigator. Japan and Singapore remained in the twenty countries with the highest publication density globally. Taiwan was ranked sixth before correction, but its rank could not be corrected as there was no data provided by the WHO.

The most common medical specialties of FAs publishing on SIH in South Korea were neurosurgery and anesthesiology (each accounted for 32.6% of all physicians). In Japan, most FAs were neurosurgeons (54.7%). In Singapore 66.7% were neurologists. In Taiwan, although not corrected, 60.5% were neurologists.

#### 3.4.3 Europe

Fifty-five percent of countries in the top twenty were European. As shown in Fig. 3 and Suppl. Table 1, most publications originated from Italy, the United Kingdom (UK), France, Germany, and Switzerland. The densest European networks of SIH researchers can be found in Switzerland, Cyprus, and the UK.

In Switzerland most of the first authors were neurosurgeons (60.0%). In Cyprus, the UK, Italy, France and Germany, the majority were neurologists (50.0%, 29.6%, 41.1%, 29.0%, 42.3%).

#### 3.4.4 Middle East

Turkey was the only Middle Eastern country in the top twenty. After correction, Turkey still led the ranking, with the highest density of SIH physicians in the Middle East. However, Oman, Qatar and Morocco were included in the corrected top twenty. Most of the authors publishing on SIH in Turkey were radiologists (47.2%).

#### 3.4.5 Oceania

Australia ranked 19th. After rank correction, Australia moved up to tenth place, while New Zealand entered the top twenty in 18th place.

### 3.5 Ranking by medical center

Cedars-Sinai Medical Center in Los Angeles, CA, USA; Mayo Clinic in Rochester, MN, USA; and Duke University Hospital in Durham, NC, USA were the three medical centers with the largest numbers of FAs of SIH publications. These three centers accounted for 139 publications. The University Hospital of Bern, Switzerland was the first non-US-center to enter the ranking in fourth place (Table 2).

**Table 2:** Institutes with at least five SIH publications listed in decreasing order of number of affiliated first authors contributing to publications between 1983 and 2022.

HEALTH INSTITUTE FIRST AUTHOR	COUNTRY	COUNT OF FIRST AUTHORS
CEDARS-SINAI MEDICAL CENTER	United States of America	61
MAYO CLINIC	United States of America	52
DUKE UNIVERSITY	United States of America	26
BERN UNIVERSITY HOSPITAL	Switzerland	20
NIGUARDA CÀ GRANDA HOSPITAL	Italy	18
ISTANBUL UNIVERSITY	Turkey	16
UNIVERSITY OF CALIFORNIA SAN FRANCISCO	United States of America	15
NEUROLOGICAL INSTITUTE C. BESTA	Italy	15
ZHEJIANG UNIVERSITY SCHOOL OF MEDICINE	China	13
TAIPEI VETERANS GENERAL HOSPITAL	Taiwan Republic of China	12
UNIVERSITY OF YAMANASHI	Japan	7
NORTHWESTERN UNIVERSITY	United States of America	7
STANFORD UNIVERSITY SCHOOL OF MEDICINE	United States of America	7
NARA MEDICAL UNIVERSITY	Japan	7
UNIVERSITY OF TORONTO	Canada	6
SUNGKYUNKWAN UNIVERSITY SCHOOL OF MEDICINE	South Korea	6
UNIVERSITY OF ULSAN COLLEGE OF MEDICINE	South Korea	5
UNIVERSITY OF WASHINGTON	United States of America	5
TAICHUNG VETERANS GENERAL HOSPITAL	Taiwan Republic of China	5
TRAKYA UNIVERSITY FACULTY OF MEDICINE	Turkey	5
AOR SAN CARLO	Italy	5
FRENCHAY HOSPITAL	United Kingdom	5
UNIVERSITY MEDICAL CENTER FREIBURG	Germany	5

### 3.6 Analysis per publication

#### 3.6.1 Ranking: most cited papers

The most cited publications are shown in Table 3.

**Table 3:** The ten most cited articles on SIH in descending order with names of first authors.

ARTICLE TITLE	NAME OF FIRST AUTHOR	NO. OF CITATIONS
Spontaneous spinal cerebrospinal fluid leaks and intracranial hypotension	W I Schievink, 2006 (Schievink, 2006)	226
Patterns of contrast enhancement in the brain and meninges	J G Smirniotopoulos, 2007 (Jg et al., 2007)	124
Spontaneous cerebrospinal fluid leaks: from intracranial hypotension to cerebrospinal fluid hypovolemia--evolution of a concept	B Mokri, 1999 (Mokri, 1999)	74
Spontaneous low pressure, low CSF volume headaches: spontaneous CSF leaks	B Mokri, 2013 (Mokri, 2013)	71
Spontaneous spinal cerebrospinal fluid leaks and intracranial hypotension	W I Schievink et al., 1996 (Schievink et al., 1996)	70
Dural enhancement and cerebral displacement secondary to intracranial hypotension	R A Fishman, 1993 (Fishman and Dillon, 1993)	68
Diagnostic criteria for spontaneous spinal CSF leaks and intracranial hypotension	W I Schievink et al., 2008 (Schievink et al., 2008)	63
Spontaneous intracranial hypotension: report of two cases and review of the literature	T A Rando, 1992 (Rando and Fishman, 1992)	58
Misdiagnosis of spontaneous intracranial hypotension	W I Schievink, 2003 (Schievink, 2003)	55
Thunderclap headache	T J Schwedt et al., 2006 (Schwedt et al., 2006)	48

#### 3.6.2 Ranking: journals that carried the highest numbers of articles on SIH

The journals that published articles on SIH were sorted and ranked according to the number of papers on SIH (Table 4). Those that published more articles on SIH appear higher up in the Table. Most publications appeared in *Headache: The Journal of Head and Face Pain* (American Headache Society, Mount Royal, NJ, USA).

**Table 4:** The twenty journals that carried the highest numbers of articles on SIH listed with their impact factor.

PUBLICATION ORGAN	COUNT OF ARTICLES ON SIH PUBLISHED	JOURNAL IMPACT FACTOR
*Headache*	59	5.311
*AM J NEURORADIOL*	43	4.966
*J NEUROSURG*	40	5.408
*NEUROLOGY*	39	11.8
*CEPHALALGIA*	36	6.075
*WORLD NEUROSURG*	23	2.21
*NEUROL SCI*	22	3.83
*SURG NEUROL INT*	18	N/A
*NEUROSURGERY*	17	5.315
*CLIN NEUROL NEUROSUR*	16	1.885
*J NEUROSURG-SPINE*	16	3.467
*J CLIN NEUROSCI*	15	2.116
*BMJ CASE REP*	14	N/A
*CAN J NEUROL SCI*	12	2.915
*J NEUROL NEUROSURG PSYCHIATRY*	10	8.324
*NEUROL MED-CHIR*	10	2.036
*J HEADACHE PAIN*	10	8.588
*CLIN NUCL MED*	9	10.782
*NEURORADIOLOGY*	9	2.995
*ACTA NEUROL BELG*	9	2.471

#### 3.6.3 Ranking: highest impact factor (IF)

Sorting FAs by their publications in the journals with the highest Impact Factor (IF), resulted in the ranking presented in Table 5. Results are shown for journals with an IF above twelve.

**Table 5:** SIH publications sorted according to descending order of journal impact factor with first author, journal title, and publication type.

Journal Impact Factor	Name of First Author	Publication Organ	Article Title	Publication Type
176.08	W I Schievink, 2021 (Schievink, 2021)	NEW ENGL J MED	Spontaneous intracranial hypotension	RV
157.34	W I Schievink, 2006 (Schievink, 2006)	JAMA-J AM MED ASSOC	Spontaneous spinal cerebrospinal fluid leaks and intracranial hypotension	RV
93.33	S Scott, 2014 (Scott and Davenport, 2014)	BMJ-BRIT MED J	Low pressure headaches caused by spontaneous intracranial hypotension	Practical Note
59.94	A Ducros, 2015 (Ducros and Biousse, 2015)	Lancet Neurol	Headache arising from idiopathic changes in CSF pressure	RV
	T Dobrocky et al., 2022 (Dobrocky et al., 2022)	Lancet Neurol	Spontaneous intracranial hypotension: searching for the CSF leak	RV
	T J Schwedt et al., 2006 (Schwedt et al., 2006)	Lancet Neurol	Thunderclap headache	RV
29.91	A Sao-Mai Sy Tay et al., 2021 (Tay et al., 2021)	JAMA Neurol	Computed Tomography vs Heavily T2-Weighted Magnetic Resonance Myelography for the Initial Evaluation of Patients With Spontaneous Intracranial Hypotension	OA
	L D'Antona, 2021 (D'Antona et al., 2021)	JAMA Neurol	Clinical Presentation, Investigation Findings, and Treatment Outcomes of Spontaneous Intracranial Hypotension Syndrome: A Systematic Review and Meta-analysis	Meta-Analysis
	M Herwerth et al., 2016 (Herwerth et al., 2016)	JAMA Neurol	Spontaneous Cerebrospinal Fluid Leak With Venous Engorgement Mimicking a Contrast-Enhancing Cervical Mass	CR
	T Dobrocky et al., 2019 (Dobrocky et al., 2019)	JAMA Neurol	Assessing Spinal Cerebrospinal Fluid Leaks in Spontaneous Intracranial Hypotension With a Scoring System Based on Brain Magnetic Resonance Imaging Findings	OA
	V N Shah, 2020 (Shah and Dillon, 2020)	JAMA Neurol	Spontaneous Intracranial Hypotension With Brain Sagging Attributable to a Cerebrospinal Fluid-Venous Fistula	CR
29.15	M D Mamlouk et al., 2021 (Mamlouk et al., 2021)	Radiology	CT-guided Fibrin Glue Occlusion of Cerebrospinal Fluid-Venous Fistulas	OA
	T Dobrocky et al., 2018 (Dobrocky et al., 2018)	Radiology	Cryptogenic Cerebrospinal Fluid Leaks in Spontaneous Intracranial Hypotension: Role of Dynamic CT Myelography	OA
	W P Dillon, 2018 (Dillon, 2018)	Radiology	Challenges in the Diagnosis and Treatment of Spontaneous Intracranial Hypotension	Editorial
15.26	J Wu et al., 2017 (Wu et al., 2017)	Brain	Factors predicting response to the first epidural blood patch in spontaneous intracranial hypotension	OA
	M Savoiardo et al., 2007 (Savoiardo et al., 2007)	Brain	Spontaneous intracranial hypotension with deep brain swelling	OA
	Y Wang et al., 2015 (Wang et al., 2015)	Brain	Cerebrospinal fluid leakage and headache after lumbar puncture: a prospective non-invasive imaging study	OA
14.28	J C Horton, 1994 (Horton and Fishman, 1994)	Ophthalmology	Neurovisual findings in the syndrome of spontaneous intracranial hypotension from dural cerebrospinal fluid leak	CR
14.04	I Marie et al., 2012 (Marie et al., 2012)	QJM-INT J MED	Spontaneous intracranial hypotension syndrome in systemic sclerosis	CR
12.89	A Cohen, 2004 (Cohen and Jesuthasan, 2004)	Anaesthesia	Blind’ epidural blood patch for spontaneous intracranial hypotension	CR
	R Thomas, 2012 (Thomas and Thanthulage, 2012)	Anaesthesia	Intracranial hypotension headache after uncomplicated caudal epidural injection	CR

RV = Review; OA = Original Article; CR = Case Report.

*Impact factors below twelve are not shown.

## 4 Discussion

The data collected in this study confirms the exponential increase in SIH-related research. Articles on the topic date back over 70 years [9,17] and there has been a surge in publications over the past 40 years. There was an impressive increase in the average annual growth rate of 12.7% during the period from 1983 to 2021. The number of SIH patients reported has increased from single individuals to more than 4000 per year (2020). Of the many articles published on the topic, most were CR.

Articles originated from more than 50 different countries. However, more than a third of all publications originated from the USA, which accounted for most publications followed by Japan and Italy. Although, the USA was responsible for 25% of all publications in 2001, 20 years later, in 2021, almost every second publication on SIH stems from an author affiliated with an institution in the USA. The highest density of SIH researchers was observed in South Korea, Canada, and Switzerland. The “most productive medical centers”, however, are based in the USA.

Neurology, neurosurgery, and radiology were the most common specialties of FAs globally. While in Japan, the USA and Switzerland it was mostly neurosurgeons who were involved in SIH research, South Korea had equal amounts of anesthesiologists contributing. Most SIH publications from European countries had neurologists as FAs. Turkey had only radiologists.

### 4.1 Findings in context

The number of publications on SIH has increased exponentially in recent decades. To avoid bias, however, it is crucial to view this rate in context of the average annual growth rate in other science domains, which is 4–5% over the same period (Bornmann et al., 2021). Given this caveat, it can be concluded that the number of publications on SIH has increased disproportionately compared to the scientific literature in general.

A potential factor contributing to the increase in publications might be the shift in focus towards research on SIH. Many medical centers now have specialized teams studying this disease and publishing guidelines (Cheema et al., 2023). There are also international organizations offering support on the topic (Abstracts, 2022). Since the publication of two frequently cited reviews, SIH has gained even more attention (Schievink, 2021; Scott and Davenport, 2014; Ducros and Biousse, 2015; Dobrocky et al., 2022; Bornmann et al., 2021).

Ranking the publications might itself be controversial as there is no direct causality between the quality of an article and the number of citations. Some publications are cited significantly more often than others. One reason might be the usefulness of a specific publication to the community. Garfield Garfield and Wallin (2005) proposed that, as the number of citations increases, the possibility of the paper becoming of more practical use to the respective scientific community increases as well. Furthermore, it is important to view our results in the context of the ever-expanding global publication landscape (Johnson et al.).

While many articles are being published on SIH, most of them are CR. We assume that because research is increasing and meetings on SIH are being held globally, SIH has become more of a “hot topic” than ever and is increasingly included in the differential diagnosis list of neurologists, neuroradiologists, and neurosurgeons. Thus, more cases are being diagnosed and more cases are being reported.

Since the disease is quite new in terms of publications and guidelines, everyone involved might be eager to participate in the search for new pathophysiological interrelationships.

It is noteworthy, however, that many countries have published only a few articles on SIH and some populous countries have produced no publications on SIH at all. It is very plausible that the diagnosis is made too rarely in these countries.

Interestingly, the distribution of medical specialties of SIH researchers is not the same in every country. We can only speculate about possible reasons for these differences. One plausible hypothesis is that the investigators who originally diagnosed SIH were more likely to have had a surgical background, as the initial diagnosis of SIH may have been made clinically. After the discovery of SIH by surgeons, physicians with non-surgical specialties may have increasingly become involved in the study of SIH. In any case, it is significant that there is a great variability of specialties, and that interdisciplinary collaborations and teamwork are important.

### 4.2 Strengths and limitations

Although research on SIH has been changing significantly in recent years, no comprehensive bibliometric analysis has yet been conducted. Our work includes a complete survey of the publications on SIH since the first publication on PubMed. All publications included were individually screened to ensure high data quality. As a university hospital intensively engaged in interdisciplinary research in the field of SIH, we were able to collaborate with other departments and optimize the design of our study.

Nevertheless, our analysis has some limitations: While PubMed is a widely used platform, it is likely that some articles on SIH were outside the scope of our research. There are two reasons for this: First, we only looked at digitally available papers. While some articles were published before 1983, we did not search any physical libraries. Secondly, only articles on PubMed were studied. We consider PubMed to be the most popular library for medical publications. Therefore, we also assume that it is the most comprehensive. Nevertheless, some publications might have been published exclusively on other platforms and thus would not have been included in our research.

We excluded non-English articles, which could have led to some articles being omitted. However, as English is widely used in science, we believe this bias to be negligible. Besides this, not every journal has a Journal Impact Factor. Only 715 of the 974 articles could be linked with journals for which impact factors were available (731 of articles for the LAs ranking).

Even though most countries and regions are represented in the WHO, data on physician density are missing for some countries. This is the case for Taiwan and thus the rank of this country could not be corrected.

## 5 Conclusions

Research and publications about SIH have increased exponentially over the past 40 years. With new treatment options awaiting discovery, research on SIH shows great promise. In parallel with the growth in publications, there is a growing number of SIH researchers. This paves the way for more international cooperation on research, treatment, and patient education. With this article we would like to encourage large-scale collaborations and we firmly believe that research on SIH can be both facilitated and nurtured. By providing information on the most active centers and researchers, new options for transnational partnerships arise.

## Consent for publication

All authors revised the manuscript and approved the version to be published.

## Funding

This research did not receive any specific grant from funding agencies in the public, commercial, or not-for-profit sectors.

## Declaration of competing interest

The authors declare that they have no known competing financial interests or personal relationships that could have appeared to influence the work reported in this paper.

## References

[bib1] Abstracts (2022). Spinal CSF leak found n.d. https://spinalcsfleak.org/resources/publication-abstracts/abstracts-2022/.

[bib2] Bornmann L., Haunschild R., Mutz R. (2021). Growth rates of modern science: a latent piecewise growth curve approach to model publication numbers from established and new literature databases. Humanit Soc Sci Commun.

[bib3] Brinjikji W., Savastano L.E., Atkinson J.L.D., Garza I., Farb R., Cutsforth-Gregory J.K. (2021). A novel endovascular therapy for CSF hypotension secondary to CSF-venous fistulas. Am. J. Neuroradiol..

[bib4] Cheema S., Anderson J., Angus-Leppan H., Armstrong P., Butteriss D., Jones L.C. (2023). Multidisciplinary consensus guideline for the diagnosis and management of spontaneous intracranial hypotension. J. Neurol. Neurosurg. Psychiatry.

[bib5] Clarivate W. of SG. (2022). Web of science master journal list. Web Sci Group Clarivate Co.

[bib6] Cohen A., Jesuthasan M. (2004). “Blind” epidural blood patch for spontaneous intracranial hypotension. Anaesthesia.

[bib7] Dillon W.P. (2018). Challenges in the diagnosis and treatment of spontaneous intracranial hypotension. Radiology.

[bib8] DISCUSSION ON INTRACRANIAL HYPOTENSION [Abstract] (1948). Proc. Roy. Soc. Med..

[bib9] Dobrocky T., Mosimann P.J., Zibold F., Mordasini P., Raabe A., Ulrich C.T. (2018). Cryptogenic cerebrospinal fluid leaks in spontaneous intracranial hypotension: role of dynamic CT myelography. Radiology.

[bib10] Dobrocky T., Grunder L., Breiding P.S., Branca M., Limacher A., Mosimann P.J. (2019). Assessing spinal cerebrospinal fluid leaks in spontaneous intracranial hypotension with a scoring system based on brain magnetic resonance imaging findings. JAMA Neurol..

[bib11] Dobrocky T., Nicholson P., Häni L., Mordasini P., Krings T., Brinjikji W. (2022). Spontaneous intracranial hypotension: searching for the CSF leak. Lancet Neurol..

[bib12] Ducros A., Biousse V. (2015). Headache arising from idiopathic changes in CSF pressure. Lancet Neurol..

[bib13] D'Antona L., Jaime Merchan M.A., Vassiliou A., Watkins L.D., Davagnanam I., Toma A.K. (2021). Clinical presentation, investigation findings, and treatment outcomes of spontaneous intracranial hypotension syndrome: a systematic review and meta-analysis. JAMA Neurol..

[bib14] Fichtner J., Ulrich C.T., Fung C., Knüppel C., Veitweber M., Jilch A. (2016). Management of spontaneous intracranial hypotension – transorbital ultrasound as discriminator. J. Neurol. Neurosurg. Psychiatry.

[bib15] Fishman R.A., Dillon W.P. (1993). Dural enhancement and cerebral displacement secondary to intracranial hypotension. Neurology.

[bib16] Garfield E. Is Citation Analysis a Legitimate Evaluation Tool? n.d.:17.

[bib17] Herwerth M., Prothmann S., Kreiser K., Hemmer B., Ploner M. (2016). Spontaneous cerebrospinal fluid leak with venous engorgement mimicking a contrast-enhancing cervical mass. JAMA Neurol..

[bib18] Hoffmann J., Goadsby P.J. (2013). Update on intracranial hypertension and hypotension. Curr. Opin. Neurol..

[bib19] Horton J.C., Fishman R.A. (1994). Neurovisual findings in the syndrome of spontaneous intracranial hypotension from dural cerebrospinal fluid leak. Ophthalmology.

[bib20] Jesse C.M., Häni L., Fung C., Ulrich C.T., Schär R.T., Dobrocky T. (2022). The impact of spontaneous intracranial hypotension on social life and health-related quality of life. J. Neurol..

[bib21] Jesse C.M., Schär R.T., Goldberg J., Fung C., Ulrich C.T., Dobrocky T. (2024). Patient-reported symptomatology and its course in spontaneous intracranial hypotension – beware of a chameleon. Clin. Neurol. Neurosurg..

[bib22] Jg S., Fm M., Ej R., Jh R., Jw S. (2007). Patterns of contrast enhancement in the brain and meninges. Radiogr Rev Publ Radiol Soc N Am Inc.

[bib23] Rob Johnson, Anthony Watkinson, Michael Mabe. The STM Report: an Overview of Scientific and Scholarly Journal Publishing. 1968 - 2018. n.d.

[bib24] Mamlouk M.D., Shen P.Y., Sedrak M.F., Dillon W.P. (2021). CT-Guided fibrin glue occlusion of cerebrospinal fluid-venous fistulas. Radiology.

[bib25] Marie I., Tollard E., Gerardin E., Guegan-Massardier E. (2012). Spontaneous intracranial hypotension syndrome in systemic sclerosis. QJM Mon J Assoc Physicians.

[bib26] Mokri B. (1999). Spontaneous cerebrospinal fluid leaks: from intracranial hypotension to cerebrospinal fluid hypovolemia--evolution of a concept. Mayo Clin. Proc..

[bib27] Mokri B. (2013). Spontaneous low pressure, low CSF volume headaches: spontaneous CSF leaks. Headache.

[bib28] Page M.J., McKenzie J.E., Bossuyt P.M., Boutron I., Hoffmann T.C., Mulrow C.D. (2021). The PRISMA 2020 statement: an updated guideline for reporting systematic reviews. Syst. Rev..

[bib29] Pinto M.J., Braz L., Fonseca J., Pereira P., Trigo Barbosa P., Gomes A. (2023). [Guidelines for the diagnosis and treatment of spontaneous intracranial hypotension]. Acta Med. Port..

[bib30] Rando T.A., Fishman R.A. (1992). Spontaneous intracranial hypotension: report of two cases and review of the literature. Neurology.

[bib31] Raskin N.H. (1990). Lumbar puncture headache: a review. Headache J. Head Face Pain.

[bib32] Relationship of bern Score, spinal elastance, and opening pressure in patients with spontaneous intracranial hypotension | neurology n.d. https://n.neurology.org/content/100/22/e2237.

[bib33] Roser M., Ritchie H., Ortiz-Ospina E., Rodés-Guirao L. (2021). https://ourworldindata.org/world-population-growth.

[bib34] Savoiardo M., Minati L., Farina L., De Simone T., Aquino D., Mea E. (2007). Spontaneous intracranial hypotension with deep brain swelling. Brain J Neurol.

[bib35] Schievink W.I. (2000). Spontaneous spinal cerebrospinal fluid leaks: a review. Neurosurg. Focus.

[bib36] Schievink W.I. (2003). Misdiagnosis of spontaneous intracranial hypotension. Arch. Neurol..

[bib37] Schievink W.I. (2006). Spontaneous spinal cerebrospinal fluid leaks and intracranial hypotension. JAMA.

[bib38] Schievink W.I. (2021). Spontaneous intracranial hypotension. N. Engl. J. Med..

[bib39] Schievink W.I., Meyer F.B., Atkinson J.L., Mokri B. (1996). Spontaneous spinal cerebrospinal fluid leaks and intracranial hypotension. J. Neurosurg..

[bib40] Schievink W.I., Maya M.M., Moser F., Tourje J., Torbati S. (2007). Frequency of spontaneous intracranial hypotension in the emergency department. J. Headache Pain.

[bib41] Schievink W.I., Maya M.M., Louy C., Moser F.G., Tourje J. (2008). Diagnostic criteria for spontaneous spinal CSF leaks and intracranial hypotension. AJNR Am J Neuroradiol.

[bib42] Schievink W.I., Maya M.M., Moser F.G., Simon P., Nuño M. (2022). Incidence of spontaneous intracranial hypotension in a community: beverly Hills, California, 2006-2020. Cephalalgia Int J Headache.

[bib43] Schwedt T.J., Matharu M.S., Dodick D.W. (2006). Thunderclap headache. Lancet Neurol..

[bib44] Scott S., Davenport R. (2014). Low pressure headaches caused by spontaneous intracranial hypotension. BMJ.

[bib45] Shah V.N., Dillon W.P. (2020). Spontaneous intracranial hypotension with brain sagging attributable to a cerebrospinal fluid-venous fistula. JAMA Neurol..

[bib46] Tay A.S.-M.S., Maya M., Moser F.G., Nuño M., Schievink W.I. (2021). Computed tomography vs heavily T2-weighted magnetic resonance myelography for the initial evaluation of patients with spontaneous intracranial hypotension. JAMA Neurol..

[bib47] Thomas R., Thanthulage S. (2012). Intracranial hypotension headache after uncomplicated caudal epidural injection. Anaesthesia.

[bib48] Wallin J.A. (2005). Bibliometric methods: pitfalls and possibilities. Basic Clin. Pharmacol. Toxicol..

[bib49] Wang Y.-F., Fuh J.-L., Lirng J.-F., Chen S.-P., Hseu S.-S., Wu J.-C. (2015). Cerebrospinal fluid leakage and headache after lumbar puncture: a prospective non-invasive imaging study. Brain J Neurol.

[bib50] World Health Organization (WHO) (2023). Medical doctors (per 10 000 population). https://www.who.int/data/gho/data/indicators/indicator-details/GHO/medical-doctors-(per-10-000-population.

[bib51] Wu J.-W., Hseu S.-S., Fuh J.-L., Lirng J.-F., Wang Y.-F., Chen W.-T. (2017). Factors predicting response to the first epidural blood patch in spontaneous intracranial hypotension. Brain J Neurol.

