# Supplementary material for: Research productivity on spontaneous intracranial hypotension: A bibliometric analysis
Source: Brain Spine. 2024 Aug 30;4:103324. doi: 10.1016/j.bas.2024.103324 (PMC11402320; doi:10.1016/j.bas.2024.103324)
Supplement: Multimedia component 1 [file mmc1.docx]

Suppl. Table 1. List of countries according to their count of the number of times first authors reported being affiliated to an institution in the listed country*

| Country of First Author | Count of Publications First Author | Rank | Percentage of Publications |
| --- | --- | --- | --- |
| United States of America | 329 | 1 | 33.8 |
| Japan | 106 | 2 | 10.9 |
| Italy | 73 | 3 | 7.5 |
| United Kingdom | 54 | 4 | 5.5 |
| South Korea | 46 | 5 | 4.7 |
| Taiwan Republic of China | 43 | 6 | 4.4 |
| Turkey | 36 | 7 | 3.7 |
| China | 36 | 8 | 3.7 |
| Canada | 32 | 9 | 3.3 |
| France | 31 | 10 | 3.2 |
| India | 27 | 11 | 2.8 |
| Germany | 26 | 12 | 2.7 |
| Switzerland | 25 | 13 | 2.6 |
| Australia | 21 | 14 | 2.2 |
| Spain | 13 | 15 | 1.3 |
| The Netherlands | 6 | 16 | 0.6 |
| Portugal | 5 | 17 | 0.5 |
| Belgium | 5 | 18 | 0.5 |
| Poland | 5 | 19 | 0.5 |
| Ireland | 4 | 20 | 0.4 |

*Countries from which fewer than three publications were identified are not shown.
