# Supplementary material for: Research productivity on spontaneous intracranial hypotension: A bibliometric analysis
Source: Brain Spine. 2024 Aug 30;4:103324. doi: 10.1016/j.bas.2024.103324 (PMC11402320; doi:10.1016/j.bas.2024.103324)
Supplement: Multimedia component 2 [file mmc2.docx]

Suppl. Table 2. Countries Ranked According to their Density of SIH Researchers from Lowest to Highest*^†^

| Country of First Author | No of First Authors | Corrected Rank | Publications per 100 000 Population |
| --- | --- | --- | --- |
| South Korea | 44 | 1 | 37.86 |
| Canada | 32 | 2 | 34.04 |
| Switzerland | 13 | 3 | 33.66 |
| Cyprus | 2 | 4 | 29.91 |
| Turkey | 29 | 5 | 27.13 |
| Japan | 86 | 6 | 26.40 |
| United Kingdom | 51 | 7 | 25.77 |
| Oman | 2 | 8 | 22.19 |
| Singapore | 3 | 9 | 20.75 |
| Australia | 21 | 10 | 19.75 |
| Ireland | 4 | 11 | 19.75 |
| United States of America | 222 | 12 | 18.53 |
| Italy | 38 | 13 | 15.55 |
| Qatar | 1 | 14 | 14.89 |
| The Netherlands | 6 | 15 | 14.40 |
| Croatia | 2 | 16 | 14.21 |
| France | 27 | 17 | 12.59 |
| New Zealand | 2 | 18 | 11.09 |
| Portugal | 5 | 19 | 8.65 |
| Morocco | 2 | 20 | 7.37 |

* WHO’s data on density of medical doctors per 10 000 population was used.

† Densities lower than 6.15 publications per 100 000 population are not shown.
