# Supplementary material for: Research productivity on spontaneous intracranial hypotension: A bibliometric analysis
Source: Brain Spine. 2024 Aug 30;4:103324. doi: 10.1016/j.bas.2024.103324 (PMC11402320; doi:10.1016/j.bas.2024.103324)
Supplement: Multimedia component 3 [file mmc3.docx]

Suppl. Table 3. Overview of the Distribution of Medical Specialties in the Fifteen Countries with the Highest Number of Publications on SIH

| Country of Affiliation of First Author | Specialty of First Author | number of first authors reporting an affiliation in the country specified |
| --- | --- | --- |
| United states of america (USA) | NS | 110 |
|  | R | 81 |
|  | N | 53 |
|  | O | 32 |
|  | OTO | 22 |
|  | AN | 21 |
|  | OPH | 7 |
|  | IM | 2 |
|  | Neurosciences | 1 |
| USA Total |  | **329** |
| Japan | NS | 58 |
|  | N | 12 |
|  | R | 11 |
|  | AN | 9 |
|  | O | 7 |
|  | OTO | 3 |
|  | IM | 2 |
|  | OPH | 2 |
|  | Orthopedic Surgery | 2 |
| Japan Total |  | **106** |
| Italy | N | 30 |
|  | Neurosciences | 18 |
|  | O | 7 |
|  | NS | 7 |
|  | R | 6 |
|  | OTO | 2 |
|  | AN | 2 |
|  | IM | 1 |
| Italy Total |  | **73** |
| United Kingdom (UK) | N | 16 |
|  | R | 12 |
|  | O | 8 |
|  | NS | 7 |
|  | Neurosciences | 4 |
|  | OTO | 3 |
|  | AN | 3 |
|  | OPH | 1 |
| UK Total |  | **54** |
| South Korea | AN | 15 |
|  | NS | 15 |
|  | N | 8 |
|  | R | 5 |
|  | O | 3 |
| South Korea Total |  | **46** |
| Taiwan, Republic of China (ROC) | N | 26 |
|  | R | 9 |
|  | O | 3 |
|  | AN | 3 |
|  | NS | 2 |
| Taiwan Republic of China Total |  | **43** |
| Turkey | R | 17 |
|  | N | 8 |
|  | O | 7 |
|  | NS | 3 |
|  | OTO | 1 |
| Turkey Total |  | **36** |
| China | N | 20 |
|  | AN | 6 |
|  | R | 4 |
|  | NS | 4 |
|  | O | 1 |
|  | OPH | 1 |
| China Total |  | **36** |
| Canada | N | 7 |
|  | NS | 7 |
|  | R | 6 |
|  | AN | 5 |
|  | Neurosciences | 4 |
|  | O | 2 |
|  | OTO | 1 |
| Canada Total |  | **32** |
| France | N | 9 |
|  | OTO | 6 |
|  | R | 6 |
|  | O | 4 |
|  | AN | 3 |
|  | IM | 1 |
|  | OPH | 1 |
|  | NS | 1 |
| France Total |  | **31** |
| India | N | 9 |
|  | R | 6 |
|  | O | 4 |
|  | AN | 4 |
|  | NS | 2 |
|  | Neurosciences | 1 |
|  | OTO | 1 |
| India Total |  | **27** |
| Germany | N | 11 |
|  | R | 9 |
|  | O | 2 |
|  | NS | 2 |
|  | Neurosciences | 1 |
|  | OPH | 1 |
| Germany Total |  | **26** |
| Switzerland | NS | 15 |
|  | R | 8 |
|  | Orthopedic Surgery | 2 |
| Switzerland Total |  | **25** |
| Australia | O | 6 |
|  | N | 4 |
|  | R | 3 |
|  | NS | 3 |
|  | OTO | 2 |
|  | AN | 1 |
|  | Neurosciences | 1 |
|  | OPH | 1 |
| Australia Total |  | **21** |
| Spain | N | 6 |
|  | AN | 3 |
|  | R | 2 |
|  | O | 1 |
|  | NS | 1 |
| Spain Total |  | **13** |

AN = anesthesiology; IM = internal medicine; N = neurology; NS = neurological surgery; O = others; OPH = ophthalmology; OTO = otolaryngology; R = radiology and neuroradiology
