# Supplementary material for: Research productivity on spontaneous intracranial hypotension: A bibliometric analysis
Source: Brain Spine. 2024 Aug 30;4:103324. doi: 10.1016/j.bas.2024.103324 (PMC11402320; doi:10.1016/j.bas.2024.103324)
Supplement: Multimedia component 4 [file mmc4.docx]

Suppl. Table 4. Ranking of the Most Cited First and Last Authors

| Name of First Author | Total No. of Citations | Rank |  | Name of Last Author | | Total No. of Citations | Rank |
| --- | --- | --- | --- | --- | --- | --- | --- |
| W I Schievink | 1081 | 1 |  | L Gray | 153 | | 1 |
| B Mokri | 325 | 2 |  | S Wang | | 134 | 2 |
| P G Kranz | 219 | 3 |  | J Tourje | | 127 | 3 |
| E Ferrante | 129 | 4 |  | W P Dillon | | 124 | 4 |
| J G Smirniotopoulos | 124 | 5 |  | J W Schroeder | | 124 | 5 |
| T J Schwedt | 83 | 6 |  | D W Dodick | | 106 | 6 |
| J Beck | 81 | 7 |  | B Mokri | | 93 | 7 |
| R A Fishman | 68 | 8 |  | M Marcel Maya | | 91 | 8 |
| T A Rando | 58 | 9 |  | R A Fishman | | 77 | 9 |
| Y Wang | 55 | 10 |  | A Raabe | | 73 | 10 |
