# Supplementary material for: Research productivity on spontaneous intracranial hypotension: A bibliometric analysis
Source: Brain Spine. 2024 Aug 30;4:103324. doi: 10.1016/j.bas.2024.103324 (PMC11402320; doi:10.1016/j.bas.2024.103324)
Supplement: Multimedia component 5 [file mmc5.docx]

Suppl. Table 5. Ranking of First and Last Authors According to their Publication Count

| Name of First Author | Number of Articles with Author Named as the First Author | Rank |  | Number of Articles with Author Named as the Last Author | Count of Name Last Author | Rank |
| --- | --- | --- | --- | --- | --- | --- |
| W I Schievink | 60 | 1 |  | S Wang | 12 | 1 |
| E Ferrante | 24 | 2 |  | M Marcel Maya | 10 | 2 |
| P G Kranz | 16 | 3 |  | J Beck | 10 | 2 |
| B Mokri | 9 | 4 |  | L Gray | 10 | 2 |
| S Albayram | 7 | 5 |  | T J Amrhein | 9 | 3 |
| T Dobrocky | 6 | 6 |  | W P Dillon | 8 | 4 |
| E Mea | 6 | 6 |  | G Bussone | 7 | 5 |
